# Recurrent fusions in *PLAGL1* define a distinct subset of pediatric-type supratentorial neuroepithelial tumors

**DOI:** 10.1007/s00401-021-02356-6

**Published:** 2021-08-05

**Authors:** Philipp Sievers, Sophie C. Henneken, Christina Blume, Martin Sill, Daniel Schrimpf, Damian Stichel, Konstantin Okonechnikov, David E. Reuss, Julia Benzel, Kendra K. Maaß, Marcel Kool, Dominik Sturm, Tuyu Zheng, David R. Ghasemi, Patricia Kohlhof-Meinecke, Ofelia Cruz, Mariona Suñol, Cinzia Lavarino, Viktoria Ruf, Henning B. Boldt, Mélanie Pagès, Celso Pouget, Leonille Schweizer, Mariëtte E. G. Kranendonk, Noreen Akhtar, Stephanie Bunkowski, Christine Stadelmann, Ulrich Schüller, Wolf C. Mueller, Hildegard Dohmen, Till Acker, Patrick N. Harter, Christian Mawrin, Rudi Beschorner, Sebastian Brandner, Matija Snuderl, Zied Abdullaev, Kenneth Aldape, Mark R. Gilbert, Terri S. Armstrong, David W. Ellison, David Capper, Koichi Ichimura, Guido Reifenberger, Richard G. Grundy, Nada Jabado, Lenka Krskova, Michal Zapotocky, Ales Vicha, Pascale Varlet, Pieter Wesseling, Stefan Rutkowski, Andrey Korshunov, Wolfgang Wick, Stefan M. Pfister, David T. W. Jones, Andreas von Deimling, Kristian W. Pajtler, Felix Sahm

**Affiliations:** 1 Department of Neuropathology, Institute of Pathology, University Hospital Heidelberg, Heidelberg, Germany; 2 Clinical Cooperation Unit Neuropathology, German Consortium for Translational Cancer Research (DKTK), German Cancer Research Center (DKFZ), Heidelberg, Germany; 3 Hopp Children’s Cancer Center Heidelberg (KiTZ), Heidelberg, Germany; 4 Division of Pediatric Neurooncology, German Cancer Consortium (DKTK), German Cancer Research Center (DKFZ), Heidelberg, Germany; 5 Bioinformatics and Omics Data Analytics, German Cancer Research Center (DKFZ), Heidelberg, Germany; 6 Department of Pediatric Oncology, Hematology, Immunology and Pulmonology, University Hospital Heidelberg, Heidelberg, Germany; 7 Princess Máxima Center for Pediatric Oncology, Utrecht, The Netherlands; 8 Pediatric Glioma Research Group, German Cancer Research Center (DKFZ), Heidelberg, Germany; 9 Faculty of Biosciences, Heidelberg University, 69117 Heidelberg, Germany; 10 Department of Pathology, Klinikum Stuttgart, Stuttgart, Germany; 11 Department of Pediatric Oncology, Hospital Sant Joan de Déu, Esplugues de Llobregat, Barcelona, Spain; 12 Department of Pathology, Hospital Sant Joan de Déu, Esplugues de Llobregat, Barcelona, Spain; 13 Laboratory of Molecular Oncology, Hospital Sant Joan de Déu, Esplugues de Llobregat, Barcelona, Spain; 14 Institute of Neuropathology, Ludwig-Maximilian University, Munich, Germany; 15 Department of Pathology, Odense University Hospital, Odense, Denmark; 16 Department of Clinical Research, University of Southern Denmark, Odense, Denmark; 17 Department of Neuropathology, GHU Paris Psychiatry and Neurosciences, Sainte-Anne Hospital, Paris, France; 18 Laboratory of Translational Research in Pediatric Oncology, SIREDO, INSERM U830, Institut Curie, Paris Sciences Lettres University, Paris, France; 19 Department of Pathology, CHRU, Nancy, France; 20 Charité - Universitätsmedizin Berlin, corporate member of Freie Universität Berlin and Humboldt-Universität zu Berlin, Institute of Neuropathology, Berlin, Germany; 21 German Cancer Consortium (DKTK), Partner Site Berlin, German Cancer Research Center (DKFZ), Heidelberg, Germany; 22 Department of Pathology, University Medical Center Utrecht, Utrecht, The Netherlands; 23 Manchester Royal Infirmary, Manchester University NHS Foundation Trust, Manchester, UK; 24 Shaukat Khanum Memorial Cancer Hospital and Research Centre, Lahore, Pakistan; 25 Institute for Neuropathology, University Medical Centre Göttingen, Göttingen, Germany; 26 Institute of Neuropathology, University Medical Center Hamburg-Eppendorf, Hamburg, Germany; 27 Department of Pediatric Hematology and Oncology, University Medical Center Hamburg-Eppendorf, Hamburg, Germany; 28 Research Institute Children’s Cancer Center Hamburg, Hamburg, Germany; 29 Paul-Flechsig Institute of Neuropathology, University Hospital and Faculty of Medicine, Leipzig, Germany; 30 Institute of Neuropathology, University of Giessen, Giessen, Germany; 31 Frankfurt Cancer Institute (FCI), University Hospital, Goethe University Frankfurt am Main, Frankfurt am Main, Germany; 32 Institute of Neurology (Edinger-Institute), University Hospital, Goethe University Frankfurt am Main, Frankfurt am Main, Germany; 33 German Cancer Consortium (DKTK), Partner site Frankfurt/Mainz, Frankfurt am Main, Germany; 34 German Cancer Research Center (DKFZ), Heidelberg, Germany; 35 Department of Neuropathology, Otto-Von-Guericke University, Magdeburg, Germany; 36 Department of Neuropathology, University of Tübingen, Tübingen, Germany; 37 Division of Neuropathology, National Hospital for Neurology and Neurosurgery, University College London Hospitals NHS Foundation Trust, Queen Square, London, UK; 38 Department of Neurodegenerative Disease, UCL Queen Square Institute of Neurology, Queen Square, London, UK; 39 Department of Pathology, NYU Langone Medical Center, New York, NY USA; 40 Laboratory of Pathology, Center for Cancer Research, National Cancer Institute, National Institutes of Health, Bethesda, MD USA; 41 Neuro-Oncology Branch, National Cancer Institute, Bethesda, MD USA; 42 Department of Pathology, St. Jude Children’s Research Hospital, Memphis, TN USA; 43 Division of Brain Tumor Translational Research, National Cancer Center Research Institute, Chuo-ku, Tokyo, Japan; 44 Institute of Neuropathology, Heinrich Heine University, Düsseldorf, Germany; 45 German Cancer Consortium (DKTK), Partner Site Essen/Düsseldorf, Essen/Düsseldorf, Germany; 46 Children’s Brain Tumour Research Centre, University of Nottingham, Nottingham, UK; 47 Department of Human Genetics, McGill University, Montreal, QC H3A 1B1 Canada; 48 Department of Pediatrics, McGill University, Montreal, QC H4A 3J1 Canada; 49 The Research Institute of the McGill University Health Center, Montreal, QC H4A 3J1 Canada; 50 Prague Brain Tumor Research Group, Second Faculty of Medicine, Charles University and University Hospital Motol, Prague, Czech Republic; 51 Department of Pathology and Molecular Medicine, Second Faculty of Medicine, Charles University and University Hospital Motol, Prague, Czech Republic; 52 Department of Pediatric Haematology and Oncology, Second Faculty of Medicine, Charles University and University Hospital Motol, Prague, Czech Republic; 53 Department of Pathology, Amsterdam University Medical Centers, Location VUmc and Brain Tumor Center Amsterdam, Amsterdam, The Netherlands; 54 Clinical Cooperation Unit Neurooncology, German Consortium for Translational Cancer Research (DKTK), German Cancer Research Center (DKFZ), Heidelberg, Germany; 55 Department of Neurology and Neurooncology Program, National Center for Tumor Diseases, Heidelberg University Hospital, Heidelberg, Germany

**Keywords:** Neuroepithelial tumor, Supratentorial, *PLAGL1*, *EWSR1*, *FOXO1*, *EP300*, Gene fusion

## Abstract

Ependymomas encompass a heterogeneous group of central nervous system (CNS) neoplasms that occur along the entire neuroaxis. In recent years, extensive (epi-)genomic profiling efforts have identified several molecular groups of ependymoma that are characterized by distinct molecular alterations and/or patterns. Based on unsupervised visualization of a large cohort of genome-wide DNA methylation data, we identified a highly distinct group of pediatric-type tumors (*n = *40) forming a cluster separate from all established CNS tumor types, of which a high proportion were histopathologically diagnosed as ependymoma. RNA sequencing revealed recurrent fusions involving the *pleomorphic adenoma gene-like 1* (*PLAGL1*) gene in 19 of 20 of the samples analyzed, with the most common fusion being *EWSR1:PLAGL1* (*n = *13). Five tumors showed a *PLAGL1:FOXO1* fusion and one a *PLAGL1:EP300* fusion. High transcript levels of *PLAGL1* were noted in these tumors, with concurrent overexpression of the imprinted genes *H19* and *IGF2*, which are regulated by PLAGL1. Histopathological review of cases with sufficient material (*n = *16) demonstrated a broad morphological spectrum of tumors with predominant ependymoma-like features. Immunohistochemically, tumors were GFAP positive and OLIG2- and SOX10 negative. In 3/16 of the cases, a dot-like positivity for EMA was detected. All tumors in our series were located in the supratentorial compartment. Median age of the patients at the time of diagnosis was 6.2 years. Median progression-free survival was 35 months (for 11 patients with data available). In summary, our findings suggest the existence of a novel group of supratentorial neuroepithelial tumors that are characterized by recurrent *PLAGL1* fusions and enriched for pediatric patients.

**Supplementary Information:**

The online version contains supplementary material available at 10.1007/s00401-021-02356-6.

## Introduction

Ependymomas encompass a heterogeneous group of central nervous system (CNS) neoplasms that occur along the entire neuroaxis and can affect both children and adults [18]. DNA methylation and gene expression profiling efforts in recent years have identified several molecular groups of ependymoma across different anatomic sites of the CNS with distinct clinicopathological characteristics and molecular alterations or patterns [6, 7, 23–26, 40–42]. Within the supratentorial compartment, two molecularly defined types of ependymoma are characterized by recurrent gene fusions, one involving the gene *ZFTA* (formerly referred to as *C11orf95*, most frequently fused to *RELA*), and the other involving *YAP1* [3, 12, 24, 26, 43]. More recently, several reports have expanded on the spectrum of gene fusions observed in supratentorial ependymoma and ependymoma-like tumors, in particular in the pediatric setting [22, 35, 43]. Implementing these molecular markers into the WHO classification for brain tumors is of paramount importance in overcoming the challenge of histologically diverse tumor types and in increasing diagnostic accuracy. Still, many cases do not fit into the as of yet established CNS tumor types, leaving clinicians and patients with unclear or even incorrect diagnoses in further decision making.

Genome-wide DNA methylation profiling has emerged as a powerful tool for both robust classification of known CNS tumor entities and identification of novel and clinically relevant subclasses of brain tumors with characteristic alterations [5, 24]. Here, we describe a molecularly distinct subset of supratentorial neoplasms (*n = *40) with predominant ependymal appearance identified by investigation of a large cohort of DNA methylation data. These tumors harbor recurrent fusions involving the *pleomorphic adenoma gene-like 1* (*PLAGL1*) gene.

## Materials and methods

### Sample collection

Tumor samples and retrospective clinical data from 40 patients were obtained from multiple national and international collaborating centers and collected at the Department of Neuropathology of the University Hospital Heidelberg (Germany). Sample selection was based on unsupervised visualization of genome-wide DNA methylation data that revealed a molecularly distinct group of tumors (*n = *40) forming a cluster separate from all established entities. Due to the aspect of a multicenter cohort (23 different centers) including DNA methylation data that have been uploaded via the web platform https://www.molecularneuropathology.org, availability of tissue and/or clinical data was restricted for some of the cases. A proportion of data was generated in the context of the Molecular Neuropathology 2.0 study. Analysis of tissue and clinical data was performed in accordance with local ethics regulations. Clinical details of the patients are listed in Supplementary Table 1 (online resource).

### Histology and immunohistochemistry

For all cases with sufficient material (*n = *16), histological review of an H&E-stained slide was performed according to the World Health Organization (WHO) 2016 classification of tumors of the CNS [17]. Immunohistochemical staining was performed on a Ventana BenchMark ULTRA Immunostainer using the ultraView Universal DAB Detection Kit (Ventana Medical Systems, Tucson, AZ, USA). Antibodies were directed against: glial fibrillary acid protein (GFAP; Z0334, rabbit polyclonal, 1:1000 dilution, Dako Agilent, Santa Clara, CA, USA), epithelial membrane antigen (EMA; clone GP1.4, mouse monoclonal, dilution 1:1000, Thermo Fisher Scientific, Fremont, CA, USA), Sry-related HMG-BOX gene 10 (SOX10; clone EP268, rabbit monoclonal, dilution 1:100, Cell Marque Corp., Rocklin, CA, USA) and oligodendrocyte lineage transcription factor 2 (OLIG2; clone EPR2673, rabbit monoclonal, dilution 1:50, Abcam, Cambridge, UK).

### DNA methylation array processing and copy-number profiling

Genome-wide DNA methylation profiling of all samples was performed using the Infinium MethylationEPIC (EPIC) BeadChip (Illumina, San Diego, CA, USA) or Infinium HumanMethylation450 (450 k) BeadChip array (Illumina) according to the manufacturer’s instructions and as previously described [5]. Raw data were generated at the Department of Neuropathology of the University Hospital Heidelberg, the Genomics and Proteomics Core Facility of the German Cancer Research Center (DKFZ) or at respective international collaborator institutes, using both fresh-frozen and formalin-fixed paraffin-embedded (FFPE) tissue samples. All computational analyses were performed in R version 3.6.0 (R Development Core Team, 2016; https://www.R-project.org). Copy-number variation analysis from 450 k and EPIC methylation array data was performed using the conumee Bioconductor package version 1.12.0 [4]. Raw signal intensities were obtained from IDAT-files using the minfi Bioconductor package version 1.21.4. Illumina EPIC and 450 k samples were merged to a combined data set by selecting the intersection of probes present on both arrays (combineArrays function, minfi). Each sample was individually normalized by performing a background correction (shifting of the 5% percentile of negative control probe intensities to 0) and a dye-bias correction (scaling of the mean of normalization control probe intensities to 10,000) for both color channels. Subsequently, a correction for the array type (450 k/EPIC) was performed by fitting univariable, linear models to the log2-transformed intensity values (removeBatchEffect function, limma package version 3.30.11). The methylated and unmethylated signals were corrected individually. Beta-values were calculated from the retransformed intensities using an offset of 100 (as recommended by Illumina). All samples were checked for duplicates by pairwise correlation of the genotyping probes on the 450 k/EPIC array. To perform unsupervised non-linear dimension reduction, the remaining probes after standard filtering [5] were used to calculate the 1-variance weighted Pearson correlation between samples. The resulting distance matrix was used as input for t-SNE analysis (t-distributed stochastic neighbor embedding; Rtsne package version 0.13). The following non-default parameters were applied: is_distance = T, theta = 0, pca = F, max_iter = 10,000 perplexity = 20. DNA methylation sites of the *PLAGL1* imprinted region (received via http://www.humanimprints.net/#data) were visualized in a heatmap using the R-package ‘pheatmap’. Control tissue DNA methylation samples (*n = *119) as previously described [5] were used for comparison.

### RNA sequencing and analysis

RNA was extracted from FFPE tissue samples using the automated Maxwell system with the Maxwell 16 LEV RNA FFPE Kit (Promega, Madison, WI, USA), according to the manufacturer’s instructions. Transcriptome analysis using messenger RNA (mRNA) sequencing of samples for which RNA of sufficient quality and quantity was available was performed on a NextSeq 500 instrument (Illumina) as previously described [31]. This was possible for 20 tumors within the novel group and 14 *ZFTA:RELA*-fused ependymomas. In addition, a reference cohort of other glioma and glioneuronal subtypes were used for differential gene expression analysis (*YAP1:MAMLD1*-fused ependymoma (*n = *3), central neurocytoma (*n = *9), extraventricular neurocytoma (*n = *8), dysembryoplastic neuroepithelial tumor (*n = *11), papillary glioneuronal tumor (*n = *9), *KIAA1549:BRAF*-fused pilocytic astrocytoma (*n = *14), diffuse midline glioma H3 K27M mutant (*n = *14) and glioblastoma IDH wild-type (*n = *9)). Fastq files from transcriptome sequencing were used for de novo annotation of fusion transcripts using the deFuse [20] and Arriba (v1.2.0) [36] algorithms with standard parameters. All further analysis was performed in R (version 3.6.0; R Core Team, 2019) using the DESeq2 package (v1.28.1) [19]. Principal Component Analysis (PCA) was performed after variance stabilizing transformation of the count data and normalization with respect to library size, based on the selection of the top 1,000 most variable genes with relative log expression normalization. Similarities between samples were determined by computing Manhattan distances on the variance stabilized data followed by unsupervised hierarchical clustering. Differential expression testing was performed on raw count data after fitting a negative binomial model. P-values were adjusted for multiplicity by applying the Benjamini–Hochberg correction.

### Targeted next-generation DNA sequencing and mutational analysis

Genomic DNA was extracted from FFPE tumor tissue samples of 18 patients within the cohort using the automated Maxwell system with the Maxwell 16 FFPE Plus LEV DNA Purification Kit (Promega, Madison, WI, USA), according to the manufacturer’s instructions. Capture-based next-generation DNA sequencing was performed on a NextSeq 500 instrument (Illumina) as previously described [29] using a custom brain tumor panel (Agilent Technologies, Santa Clara, CA, USA) covering the entire coding and selected intronic and promoter regions of 130 genes of particular relevance in CNS tumors (Supplementary Table 2, online resource).

### Statistical analysis

Statistical analysis was performed using GraphPad Prism 9 (GraphPad Software, La Jolla, CA, USA). Data on survival could be retrospectively retrieved for eleven patients. Distribution of time to progression or recurrence (TTP) after surgery was estimated by the Kaplan–Meier method. Patients lost to follow-up are censored at date of last contact in analysis of TTP.

## Results

### DNA methylation profiling reveals a molecular distinct group of neuroepithelial tumors

**Fig. 1:**
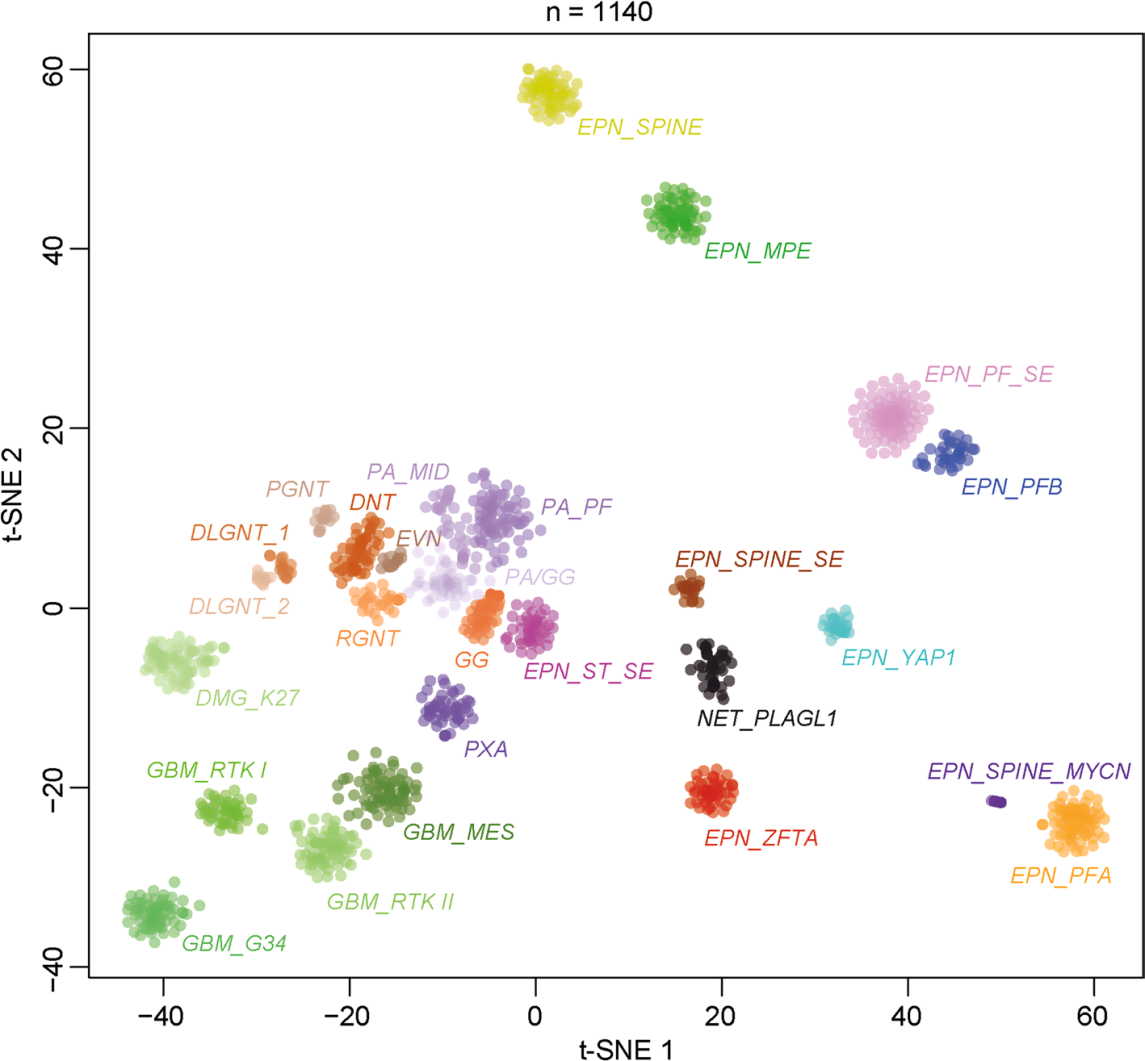
DNA methylation profiling reveals a molecular distinct group of neuroepithelial tumors. t-distributed stochastic neighbor embedding (t-SNE) analysis of DNA methylation profiles of the 40 tumors investigated (*NET_PLAGL1*) alongside 1100 selected reference samples. Reference DNA methylation classes: ependymoma posterior fossa group A (*EPN_PFA*), ependymoma posterior fossa group B (*EPN_PFB*), ependymoma spinal (*EPN_SPINE*), ependymoma with *ZFTA* fusion (*EPN_ZFTA*), ependymoma with *YAP1* fusion (*EPN_YAP1*), myxopapillary ependymoma (*EPN_MPE*), spinal ependymoma (*EPN_SPINE*), posterior fossa subependymoma (*EPN_PF_SE*), spinal subependymoma (*EPN_SPINE_SE*), supratentorial subependymoma (*EPN_ST_SE*) and spinal ependymoma with *MYCN* amplification (*EPN_SPINE_MYC*), pleomorphic xanthoastrocytoma (*PXA*), posterior fossa pilocytic astrocytoma (*PA_PF*), midline pilocytic astrocytoma (*PA_MID*), pilocytic astrocytoma and ganglioglioma (*PA/GG*), ganglioglioma (*GG*), rosette-forming glioneuronal tumor (*RGNT*), dysembryoplastic neuroepithelial tumor (*DNT*), extraventricular neurocytoma (*EVN*), papillary glioneuronal tumor (*PGNT*), diffuse leptomeningeal glioneuronal tumor subclass 1 and 2 (*DLGNT_1/2*), glioblastoma IDH wild-type subclass mesenchymal (*GBM_MES*), glioblastoma IDH wild-type subclass RTK I (*GBM_RTK I*), glioblastoma IDH wild-type subclass RTK II (*GBM_RTK II*), glioblastoma IDH wild-type H3.3 G34 mutant (*GBM_G34*) and diffuse midline glioma H3 K27M mutant (*DMG_K27*). Additional clustering analyses indicated that the PLAGL1 cohort can potentially be further subdivided into two clusters (not shown)

DNA methylation profiling has emerged as a powerful approach for robust classification of CNS neoplasms [5]. Using a screening approach based on unsupervised visualization of a large cohort of genome-wide DNA methylation data, we identified a highly distinct group of tumors (*n = *40) forming a cluster separate from all established entities of which a high proportion of tumors (19/32, 59%) were histopathologically diagnosed as ependymoma. A more focused t-SNE analysis of DNA methylation patterns of these samples alongside 1100 other well-characterized glial and glioneuronal neoplasms (reference samples included in the current version of the Heidelberg DNA methylation classifier with a calibrated score > 0.9) confirmed the distinct nature of this novel group (Fig. 1). Analysis of copy-number variations (CNVs) derived from DNA methylation array data revealed a relatively balanced profile in most of the cases, with structural aberrations on chromosome 22q (21/40, 52.5%) and 6q (19/40, 47.5%) most frequently observed (Supplementary Fig. 1a, online resource). A chromothripsis-like pattern affecting chromosomes 6 and 13 was seen in one of the samples (Supplementary Fig. 1b, online resource). In one case, a homozygous deletion of *CDKN2A/B* was detected. An integrated plot of CNVs identified in all samples is given in Supplementary Fig. 1c (online resource).

### Recurrent rearrangements involving PLAGL1 are characteristic for the novel group of neuroepithelial tumors

**Fig. 2:**
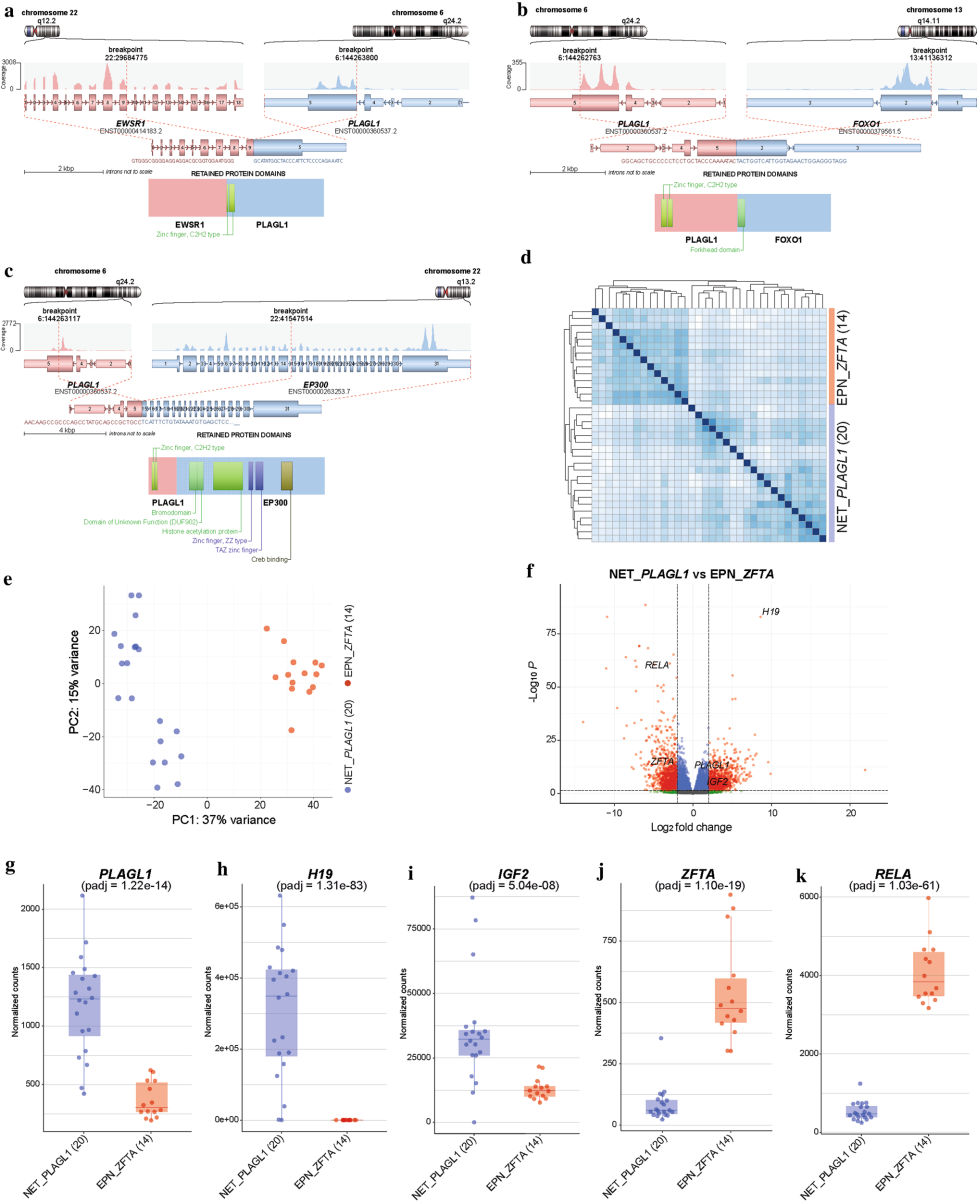
Illustration of the *PLAGL1* fusion genes and transcriptional profiling of tumors samples in the novel group (NET_*PLAGL1*). Visualization of the *PLAGL1* fusion genes detected by RNA sequencing for three selected samples. *EWSR1:PLAGL1* fusion in case #1, in which exons 1–9 of *EWSR1*, as the 5’ partner, are fused to exon 5 of *PLAGL1* (**a**), *PLAGL1:FOXO1* fusion in case #18, in which exons 1–5 of *PLAGL1* are fused to exons 2–3 of *FOXO1* as the 3’ partner (**b**), and *PLAGL1:EP300* fusion in case #19, in which exons 1–5 of *PLAGL1* are fused to exons 15–31 of *EP300* as the 3’ partner (**c**), conserving the zinc finger structure (C2H2 type) as part of the fusion products. Differences in gene expression profiles between samples in the novel group and *ZFTA:RELA*-fused ependymomas. Normalized transcript counts from samples in the novel group and *ZFTA:RELA*-fused ependymomas clustered by Pearson’s correlation coefficient (**d**) and principal component analysis (**e**). Volcano plot depicting genes differentially expressed between samples in the novel group versus *ZFTA:RELA*-fused ependymomas (**f**). *PLAGL1* (**g**), *H19* (**h**), *IGF2* (**i**), *ZFTA* (**j**), and *RELA* (**k**) expression in the novel group (*n = *20) compared to *ZFTA:RELA*-fused ependymoma samples (*n = *14)

**Fig. 3:**
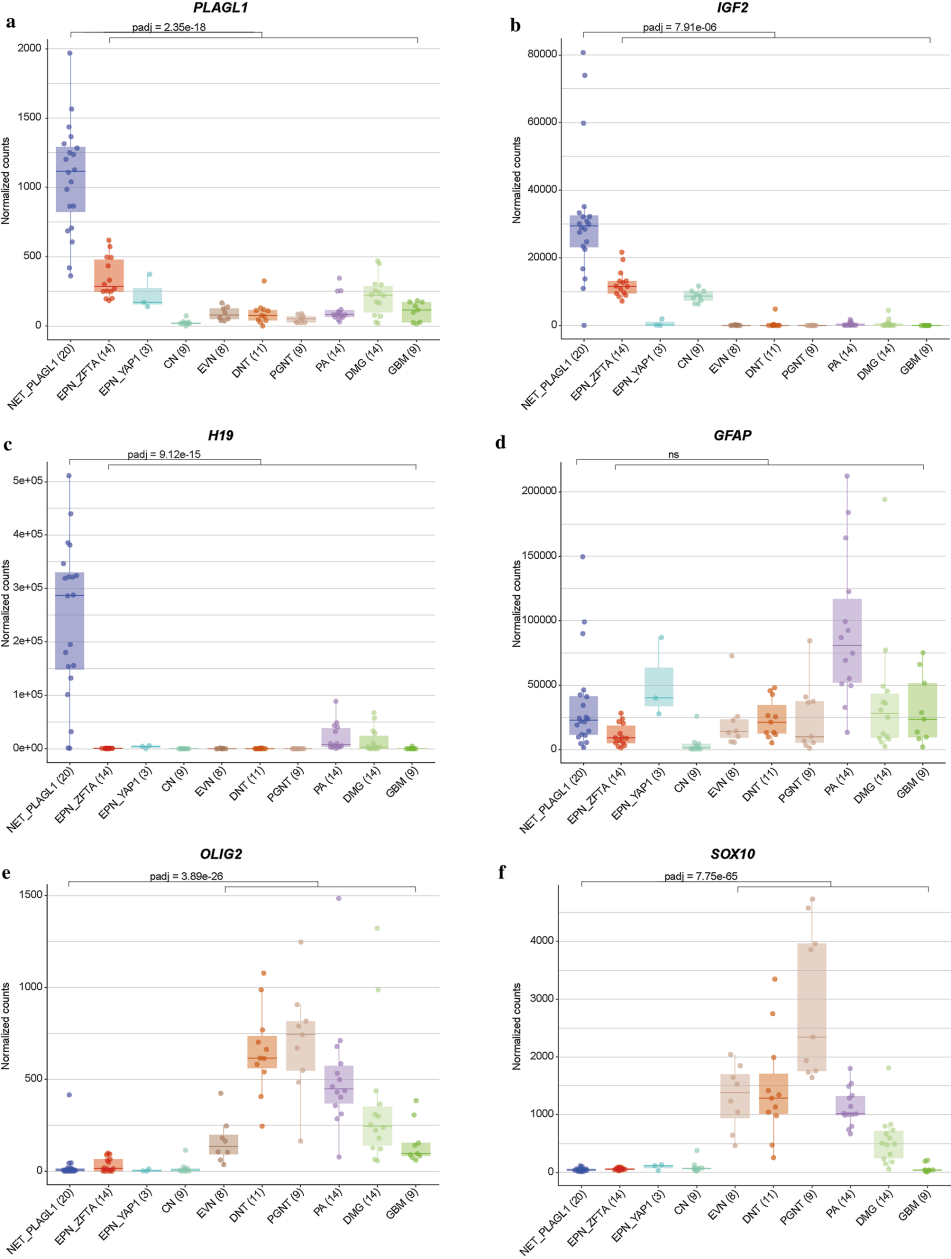
Transcriptional profiling of *PLAGL1*-altered neuroepithelial tumor. Differential gene expression analysis between samples in the novel group (NET_PLAGL1) and a reference cohort of different glial/glioneuronal tumors (*ZFTA:RELA*-fused ependymoma (EPN_ZFTA), *YAP1:MAMLD1*-fused ependymoma (EPN_YAP1), central neurocytoma (CN), extraventricular neurocytoma (EVN), dysembryoplastic neuroepithelial tumor (DNT), papillary glioneuronal tumor (PGNT), *KIAA1549:BRAF*-fused pilocytic astrocytoma (PA), diffuse midline glioma H3 K27M mutant (DMG) and glioblastoma IDH wild-type (GBM). *PLAGL1*, *IGF2* and *H19* are more highly expressed in NET_PLAGL1 cases when compared with representative glial/glioneuronal tumors (**a**–**c**). *GFAP* levels are similar compared to different glial/glioneuronal tumors (**d**). Expression of markers differentially expressed in astrocytic and in ependymal tumors revealed low *OLIG2* and *SOX10* expression in NET_PLAGL1 compared to astrocytic/glioneuronal tumors (**e**, **f**)

Since a high proportion of supratentorial ependymomas are driven by gene fusions involving *ZFTA* (*C11orf95*, most frequently fused to *RELA*) or *YAP1*, we performed mRNA sequencing of all samples with sufficient material (*n = *20). In 19/20 of the cases, a gene fusion involving *PLAGL1* was detected, conserving the zinc finger structure (C2H2 type) as part of the fusion product, with either *EWSR1* as 5’ partner or *FOXO1* or *EP300* as a 3’ partner (Fig. 2a–c). In the most common *EWSR1:PLAGL1* fusions (*n = *13), exons 1–9 or 1–8 of *EWSR1* (NM_013986), which is located on chromosome 22q12.2, were fused to exon 5 of *PLAGL1* (NM_001289039), which is found on chromosome 6q24.2. Five out of 20 cases with exons 1–5 of *PLAGL1* fused to *FOXO1* upstream of exons 2–3 (NM_0017612) were also observed. In one case, exons 1–5 of *PLAGL1* are fused to exons 15–31 of *EP300* (NM_001429). In all rearrangements, the DNA binding domain (zinc finger structure) of *PLAGL1* was retained and fused to the respective transactivation domain (TAD) of the partner gene (Fig. 2a–c). We next performed an exploratory differential gene expression analysis of tumor samples (*n = *20) within the novel group in comparison to *ZFTA:RELA*-fused ependymomas (*n = *14). Unsupervised hierarchical clustering demonstrated a clear segregation of tumor samples in comparison to *ZFTA:RELA*-fused ependymoma (Fig. 2d). These results were recapitulated by PCA of normalized transcript counts (Fig. 2e). Quantification of mRNA expression revealed that the *PLAGL1* gene itself was more highly expressed in tumors within the novel group than in *ZFTA:RELA*-fused ependymoma (adjusted *p = *1.22e − 14; Fig. 2f, g). Additionally, upregulated genes of potential interest included *H19* and *IGF2* (adjusted *p = *1.31e − 83, adjusted *p = *5.04e − 08; Fig. 2h, i), both regulated by *PLAGL1* and with known functions in the tumorigenesis of different cancers [38]. *RELA* and *ZFTA* transcript levels were upregulated in *ZFTA:RELA*-fused ependymomas (adjusted *p = *1.03e − 61 and adjusted *p = *1.10e − 19, respectively; Fig. 2j, k). Differential gene expression analysis between tumors within the novel group and a reference cohort of other glial and glioneuronal subtypes confirmed high transcript levels of *PLAGL1* (adjusted *p = *2.35e − 18), *H19* (adjusted *p = *9.12e − 15), *IGF2* (adjusted *p = *7.91e − 06) and *DLK1* (adjusted *p = *1.12e − 10) in the *PLAGL1*-fused cohort (Fig. 3a–c and Supplementary Fig. 2, online resource). Expression of particular markers differentially expressed in astrocytic and in ependymal neoplasms [10, 14, 21] revealed low *OLIG2* and *SOX10* expression (adjusted *p = *3.89e − 26 and adjusted *p = *7.75e − 65) within the novel group, with similar expression of *GFAP* (Fig. 3d–f and Supplementary Fig. 2, online resource). Moreover, the nearby imprinting control region (ICR) of *PLAGL1* showed evidence for loss of imprinting in the corresponding DNA methylation profiles (Supplementary Fig. 3, online resource). Analysis of the mutational landscape of 19/40 tumors in the novel group using targeted next-generation sequencing revealed *TERT* promoter mutations (C228T) in two of the cases (Supplementary Table 1, online resource), with no other relevant events involving putative brain tumor genes.

### Clinical characteristics and morphological features demonstrate pediatric-type tumors with ependymoma-like appearance

**Fig. 4:**
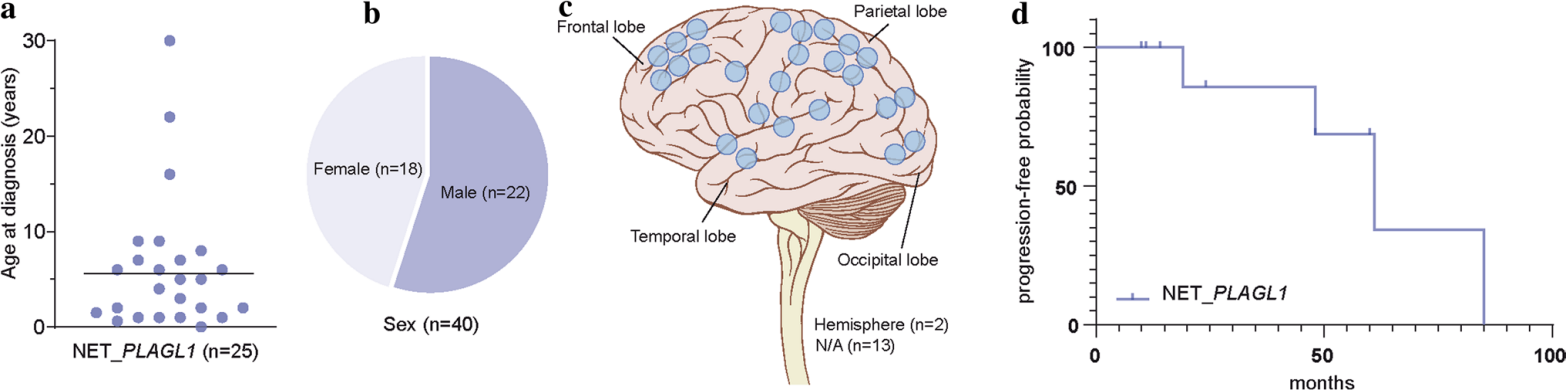
Clinical features of the investigated cohort. Age at diagnosis with the median age of 6.2 years (**a**), patient sex distribution (**b**) and distribution of tumor location (**c**). Time to progression or recurrence (TTP) of the 11 patients from the investigated cohort (NET_*PLAGL1*) for whom follow-up data were available (**d**)

**Fig. 5:**
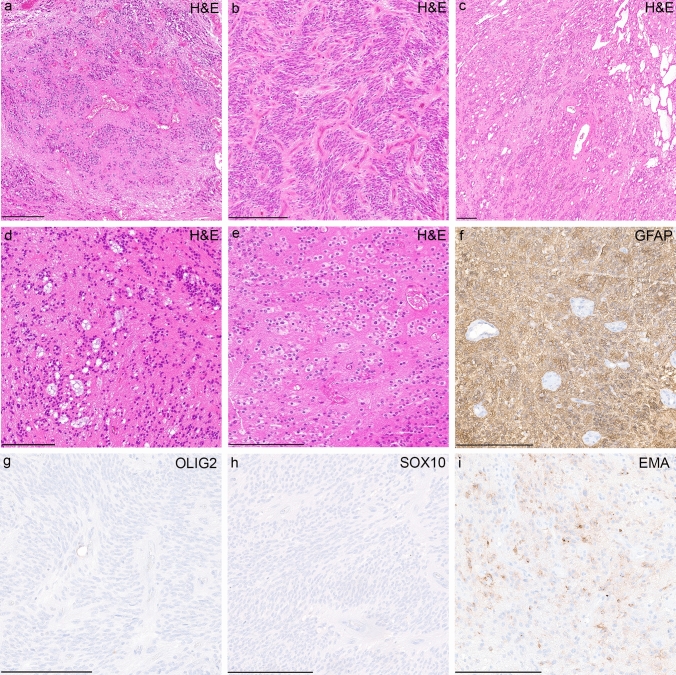
Morphological and immunohistochemical features of tumors within the cohort. Histologically, tumors shared a moderate to high increase in cellular density with mostly monomorphic, round to oval nuclei and often prominent microcystic changes (**a**–**d**). Perivascular pseudorosettes were observed in several of the cases, although very subtle in some the samples (**a**–**d**). Occasionally, tumor cells showed oligodendroglial morphology with perinuclear halos due to cytoplasmatic clearing (**e**). Immunohistochemically, tumors were GFAP positive (**f**) and OLIG2- and SOX10 negative (**g**, **h**). In 3/16 of the cases, a dot-like positivity for EMA was detected (i). Scale bars denote 200 μm

Analysis of available clinical data demonstrated that median age of the patients at the time of diagnosis was 6.2 years (*n = *25; range 0–30; with 92% of the tumors occurring in patients < 17 years of age, Fig. 4a) and the sex distribution was relatively balanced (F/M = 1:1.2, Fig. 4b). All tumors in our series were located supratentorially (Fig. 4c). The proportion of *PLAGL1*-fused tumors from all supratentorial tumors cannot yet be accurately determined. However, within the pediatric Molecular Neuropathology 2.0 study, *PLAGL1*-fused tumors account for approximately 0.7% of all supratentorial neoplasms included in the study. Outcome data were available for 11 patients. Median progression-free survival was 35 months (range 10–85 months; Fig. 4d). The initial histopathological diagnoses of the tumors within the cohort were relatively wide, although a high proportion of cases were designated as ependymoma (19/32, 59%). Other recurrent diagnoses included ‘embryonal tumor’ and different low- and high-grade gliomas (Supplementary Table 1, online resource). More detailed descriptions of the cases are given in Supplementary Table 1. A histopathological review of samples with available material (*n = *16) confirmed a relatively wide morphological spectrum of tumors with ependymoma-like features (Fig. 5a–i). Histologically, all reviewed tumors shared a moderate to high increase in cellular density in a mostly fine neurofibrillary matrix with prominent microcystic changes (Fig. 5a–d). The tumor cells typically had monomorphic, round to oval nuclei with finely dispersed chromatin and prominent nucleoli. Single cases presented more pleomorphic cells. In many cases, perivascular pseudorosettes were observed, at least focally. Two of the cases showed focal oligodendroglial morphology with perinuclear halos due to cytoplasmatic clearing (Fig. 5e). Extensive calcification was seen in a small number of tumors (*n = *3). Necrosis was not observed. Mitotic activity was generally low, with exception of two cases. Immunoreactivity for GFAP was present in all cases (Fig. 5f). The tumor cells neither expressed OLIG2 nor SOX10 (Fig. 5g, h). In 3/16 of the cases, a dot-like positivity for EMA was detected (Fig. 5i).

## Discussion

Here, we provide evidence for the pathobiological heterogeneity of neuroepithelial tumors beyond the established spectrum by reporting the existence of an epigenetically distinct group of rare pediatric-type supratentorial neoplasms with often ependymoma-like appearance that shows recurrent gene fusions involving the *PLAGL1* gene.

Our findings suggest rearrangements involving *PLAGL1*, particularly *EWSR1:PLAGL1* and *PLAGL1:FOXO1* fusions, as a molecular hallmark of this novel group of tumors. Gene fusions of *PLAGL1* with *EWSR1* have been reported exceptionally rarely in neoplasms of the CNS, including single cases of a *SMARCB1*-deficient atypical teratoid/rhabdoid tumor (AT/RT) [27] and a glioneuronal tumor, not elsewhere classified (NEC) [16]. However, in a very recent report, a *PLAGL1:EWSR1* fusion was described in a supratentorial ependymoma of a six-year-old child [44]. While *EWSR1* has long been known to be involved in gene fusions in Ewing sarcoma and several other tumor entities [34], the role of *PLAGL1* in tumorigenesis is not yet fully understood. The *PLAGL1* gene encodes a C2H2 zinc finger protein that acts as a transcription factor as well as a cofactor of other regulatory proteins, and is expressed in diverse types of human tissues amongst others in neural stem/progenitor cells and developing neuroepithelial cells [37, 39]. Although its specific role in tumorigenesis is controversial and its functions appear to depend on the cellular context, altered expression of *PLAGL1* has been linked to various types of cancer [1, 8, 32]. More recent studies provide evidence for its oncogenic function in brain tumors with overexpression of *PLAGL1* being involved in tumorigenesis of glioblastoma [9, 13] and interaction of *PLAGL* family transcription factors in *ZFTA:RELA*-fused supratentorial ependymoma [3].

In the *EWSR1:PLAGL1* fusions described here, the whole N-terminal transcriptional activation domain (TAD) of *EWSR1* is fused in-frame to the zinc finger domain (with DNA binding activity) of *PLAGL1*, very similar to other oncogenic *EWSR1* fusions, in particular rearrangements between *EWSR1* and *PATZ1* [28, 30]. This indicates aberrant recruitment of the TAD of *EWSR1* to the DNA binding domain of *PLAGL1* with subsequent downstream effects, as described for other *EWSR1* rearrangements, as the likely oncogenic function of this fusion [11]. This also fits to the increased expression of *PLAGL1* in these samples. In addition, five cases harbored a fusion between *PLAGL1* and the transcriptional factor *FOXO1*, which is a known partner in other rearrangements [2, 15]. In the *PLAGL1:FOXO1* fusion observed here, the DNA binding domain of *PLAGL1* is juxtaposed to the C-terminal TAD of *FOXO1*, which seems quite similar to *PAX3:FOXO1* rearrangements as frequently observed in alveolar rhabdomyosarcoma [15]. In a single case, *PLAGL1* was fused to *EP300*, a fusion partner known from ‘CNS tumors with *BCOR* alteration’ [33]. Additionally, upregulated genes included *H19*, *IGF2* and *DLK1*, all regulated by *PLAGL1* and with known functions in tumorigenesis of different cancers [38]. This might indicate a potential downstream effect of the fusion. However, the precise oncogenic mechanism of the EWSR1:PLAGL1, PLAGL1:FOXO1 and PLAGL1:EP300 chimeric proteins remain to be elucidated. Further studies will be needed to reveal the exact role of the fusions in these tumors.

Another important finding was the relatively wide morphological spectrum of tumors within this group. Although most tumors were originally diagnosed as ependymoma, a significant proportion of cases were designated to other entities, including different low- and high-grade tumors. Consistent with that, a histopathological review of cases with sufficient material revealed a morphologically heterogeneous group of tumors often with ependymoma-like features. A putative ependymal differentiation was further supported by differential gene expression analysis between tumors within the novel group and a reference cohort of other glial and glioneuronal tumors, that revealed low expression levels of *OLIG2* and *SOX10*, both suggested to distinguish astrocytic from ependymal tumors [10, 14, 21]. However, the absence of a unifying morphological pattern in this group of tumors underlines the relevance of molecular profiling for precise diagnosis of these CNS neoplasms. This group has not been identified as a distinct subset in previous large-scale studies due to the relatively small case numbers, broad morphology and lack of routine RNA profiling in previous cohorts, again highlighting the importance of RNA sequencing in standard brain tumor diagnostics. According to the structure of specifying ‘essential diagnostic criteria’ of the upcoming 5th edition of the WHO classification of CNS tumors, we suggest (a) the specific signature by DNA methylation profiling or (b) the combination GFAP expression and *PLAGL1* fusions as essential diagnostic criteria for these tumors.

A limitation of our study is the relatively low extent of clinical data due to diverse origins and the retrospective nature of the series, in particular patient outcome data, which allows only a rough estimation of the malignancy of the tumors within this novel group. Considering the high number of cases without sequencing data, it seems also possible that other alterations apart from the described fusions could be present, particularly in those tumors which do not show indication for a *PLAGL1* fusion in the copy-number profile. Follow-up analyses are needed to characterize this new group of CNS neoplasms in more detail.

In summary, we provide evidence for a novel group of supratentorial brain tumors and identify *PLAGL1* as a putative relevant driver in this entity. Since there is no absolutely clear indication of a particular lineage at the moment, we suggest the term ‘supratentorial neuroepithelial tumor with *PLAGL1* fusion’ to describe this novel group of tumors. However, we hope to further specify the name once additional studies provide a clearer picture of the cellular origin. These findings have immediate implications for brain tumor profiling in order to avoid incorrect diagnoses due to lack of alignment with established tumor types. *PLAGL1* fusion-positive neuroepithelial tumors should thus be included into upcoming classifications of brain tumors.

## Supplementary Information

Below is the link to the electronic supplementary material.

Supplementary file1 (PDF 503 KB)

Supplementary file2 (XLSX 21 KB)
