# Supplementary material for: Recurrent fusions in PLAGL1 define a distinct subset of pediatric-type supratentorial neuroepithelial tumors
Source: Acta Neuropathol. 2021 Aug 5;142(5):827–39. doi: 10.1007/s00401-021-02356-6 (PMC8500895; doi:10.1007/s00401-021-02356-6)
Supplement: Supplementary file 1 — Supplementary file1 (PDF 503 KB) [file 401_2021_2356_MOESM1_ESM.pdf]

Recurrent fusions in *PLAGL1* define a distinct subset of pediatric-type supratentorial neuroepithelial tumors

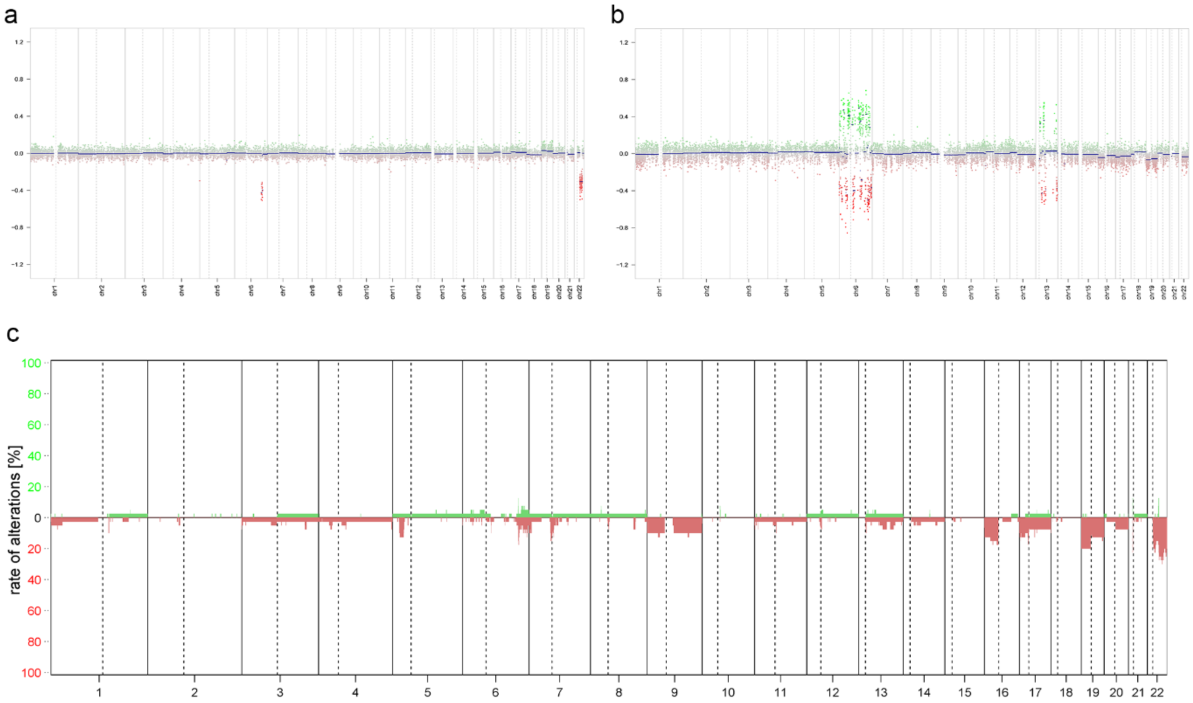

Supplementary Fig. 1 Copy-number profiles in *PLAGL1*-fused neuroepithelial tumor. Copy-number profiles derived from DNA methylation array of different tumors within the novel group showing structural alterations affecting chromosome 6q around the *PLAGL1* locus and chromosome 22q including *EWSR1* (a) as well as a chromothripsis-like pattern affecting chromosomes 6 and 13 (b). Integrated plot of copy number variations in all samples within the cohort show no recurrent chromosomal alterations besides small structural aberrations on chromosome 22q and 6q (c). The probes of the array are combined in 8000 bins (green/red dots). Gains/amplifications represent positive, losses represent negative deviations from the baseline.

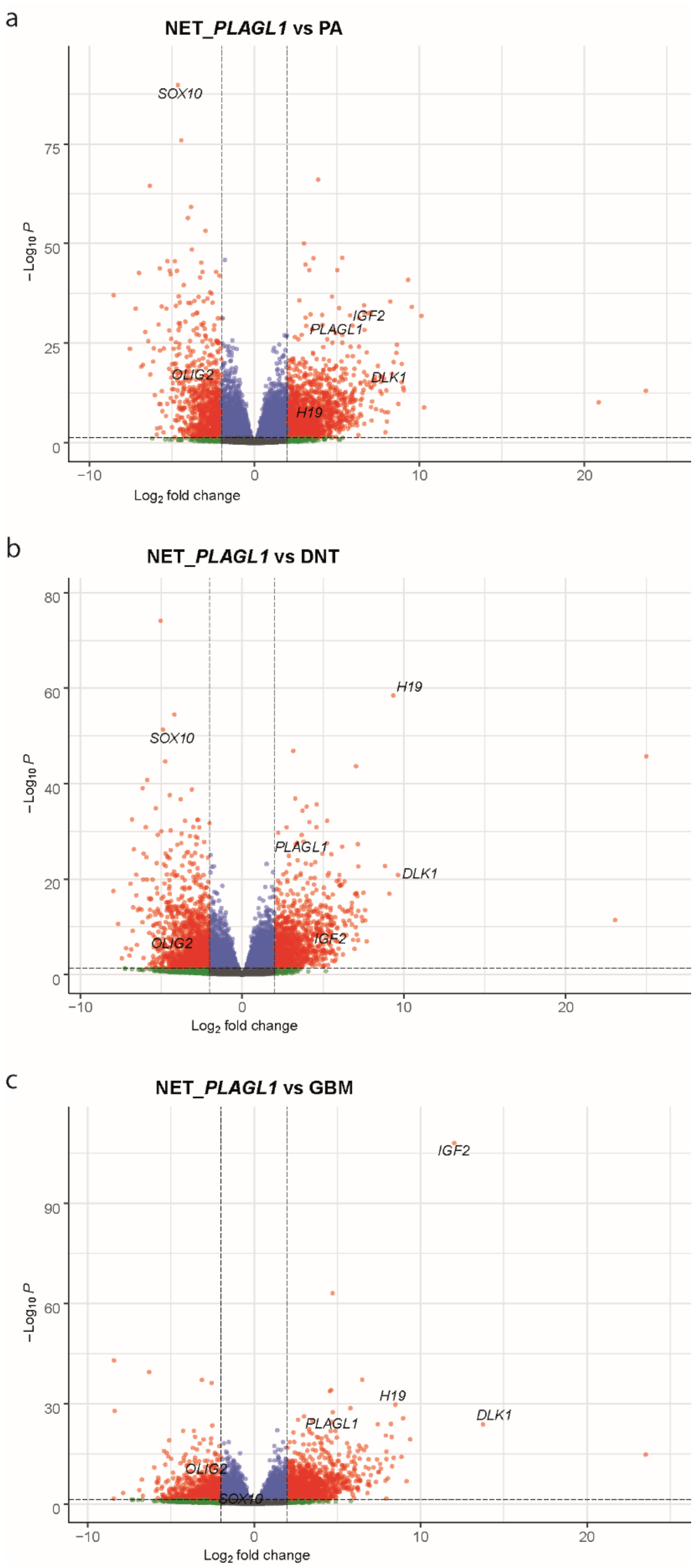

Supplementary Fig. 2 Differences in gene expression profile between *PLAGL1*-fused neuroepithelial tumors (NET_*PLAGL1*) and different glial/glioneuronal tumors. Volcano plot depicting genes differentially expressed between samples in the novel group (NET_*PLAGL1*) versus pilocytic astrocytoma (PA; a), dysembryoplastic neuroepithelial tumor (DNT; b), and glioblastoma IDH-wildtype (GBM; c). *PLAGL1* and the imprinted genes *IGF2*, *H19* and *DLK1* are more highly expressed in NET_*PLAGL1* cases when compared with representative glial/glioneuronal tumors (a-c). Low *OLIG2* and *SOX10* expression levels in NET_*PLAGL1* compared to glial/glioneuronal tumors (a-c).

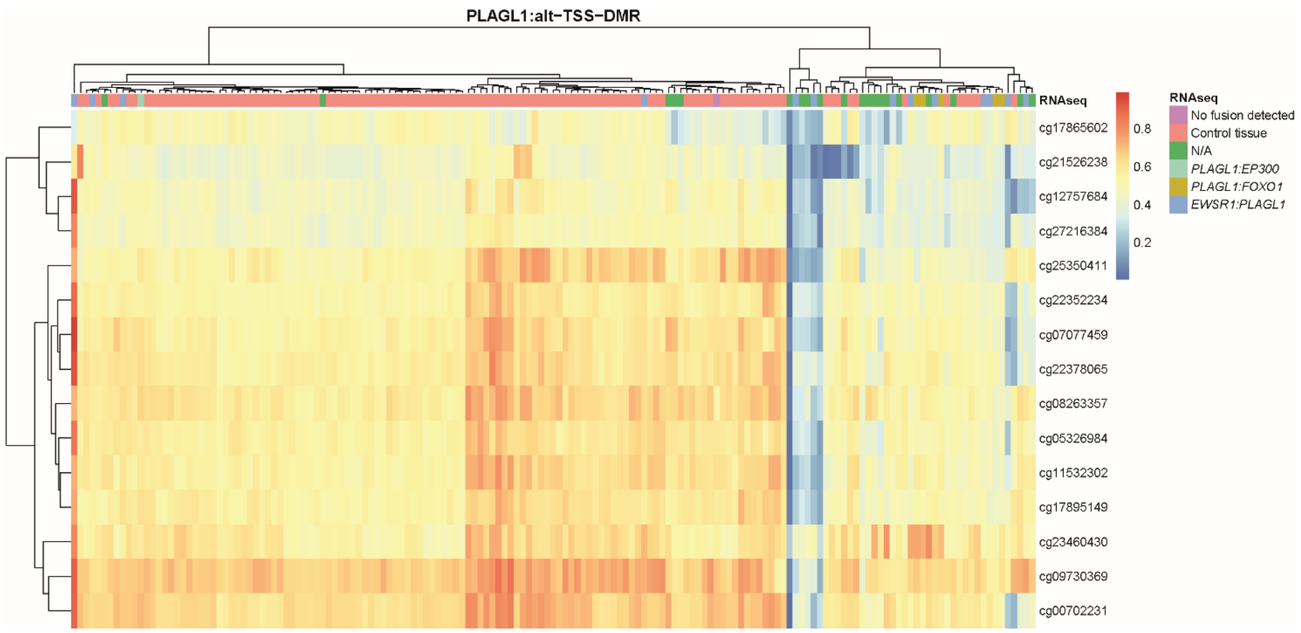

Supplementary Fig. 3 Heatmap visualization of DNA methylation sites of *PLAGL1* imprinting control region. Fifteen CpG sites of 40 PLAGL1 samples and 119 control tissue samples are shown. The hierarchical clustering of the samples separates most *PLAGL1*-fused cases from the control tissue samples and the heatmap of the methylation values displays that the CpG site in most *PLAGL1*-fused samples are slightly hypomethylated compared to the control tissue samples.
